# Clinical diversity in patients with Schnyder corneal dystrophy—a novel and known *UBIAD1* pathogenic variants

**DOI:** 10.1007/s00417-018-4075-9

**Published:** 2018-08-06

**Authors:** Anna Sarosiak, Monika Udziela, Aneta Ścieżyńska, Dominika Oziębło, Anna Wawrzynowska, Jacek P. Szaflik, Monika Ołdak

**Affiliations:** 1 Department of Histology and Embryology, Center of Biostructure Research, Medical University of Warsaw, T. Chałubińskiego 5, 02-004 Warsaw, Poland; 2 Postgraduate School of Molecular Medicine, Medical University of Warsaw, Warsaw, Poland; 3 Department of Ophthalmology, Medical University of Warsaw, Warsaw, Poland

**Keywords:** Schnyder corneal dystrophy, UBIAD1, Confocal microscopy, Optical coherent tomography, Pathogenic variant

## Abstract

**Purpose:**

Schnyder corneal dystrophy (SCD) is a rare inherited disease that leads to gradual vision loss by the deposition of lipids in the corneal stroma. The aim of this study is to report a novel pathogenic variant in the *UBIAD1* gene and present clinical and molecular findings in Polish patients with SCD.

**Methods:**

Individuals (*n* = 37) originating from four Polish SCD families were subjected for a complete ophthalmological check-up and genetic testing. Corneal changes were visualized by slit-lamp examination, anterior segment optical coherent tomography (AS-OCT), and in vivo confocal microscopy (IVCM).

**Results:**

In a proband with primarily mild SCD that progressed rapidly at the end of the fifth decade of life, a novel missense pathogenic variant in *UBIAD1* (p.Thr120Arg) was identified. The other studied SCD family represents the second family reported worldwide with the *UBIAD1* p.Asp112Asn variant. SCD in the remaining two families resulted from a frequently identified p.Asn102Ser pathogenic variant. All affected subjects presented a crystalline form of SCD. The severity of corneal changes was age-dependent, and their morphology and localization are described in detail.

**Conclusion:**

The novel p.Thr120Arg is the fourth SCD-causing variant lying within the FARM motif of the UBIAD1 protein, which underlines a high importance of this motif for SCD pathogenesis. The current study provides independent evidence for the pathogenic potential of *UBIAD1* p.Asp112Asn and new information useful for clinicians.

## Introduction

Schnyder corneal dystrophy (SCD; OMIM #121800) is a rare autosomal dominant disease classified within the group of stromal dystrophies (IC3D 2015, [1]) and caused by *UBIAD1* pathogenic variants [2–4]. SCD is characterized by progressive opacification of both corneas resulting from excessive cellular and intracellular accumulation of cholesterol and phospholipids in the corneal stroma. Lipid deposits may take form of crystals, non-crystalline stromal opacity, or *arcus lipoides* [2]. Chemical analysis of SCD corneas shows a tenfold higher content of cholesterol and fivefold higher content of lipids compared to healthy corneas [5]. SCD prevalence in the general population remains unknown. Early stages of the disease may be asymptomatic and the diagnosis may be delayed until the occurrence of a distinct haze or crystals, commonly in the second decade of life [6]. While SCD scotopic vision usually remains preserved until the late stages of the disease, photopic vision deteriorates more rapidly with the progression of corneal opacification [5, 7]. Patients complain of decreasing visual acuity (VA) and glare which is caused by light scattering from the surfaces of corneal crystals. In order to recover vision quality in advanced stages of SCD, penetrating keratoplasty (PKP) is performed. In a group of 115 individuals from 34 SCD families, 54% of patients of at least 50 years and 77% of patients aged 70 or over were subjected to PKP [5].

To date, 27 non-synonymous point alterations of the *UBIAD1* gene causative of SCD have been described [8–11]. The most frequent pathogenic variants include p.Asn102Ser, p.Gly177Glu/Arg, and p.Leu121Phe. In 2016, p.Thr103Ile, the first de novo *UBIAD1* gene pathogenic variant associated with SCD was identified [9]. UBIAD1 protein is predicted to contain ten transmembrane (TM) helices, nine of which lie within a functional prenyltransferase domain [12], which is a key part of the UBIAD1 protein enzymatic activity [10, 12–14]. TM helices emerge from the lipid bilayer into three soluble polypeptide loops. All of the so far identified pathogenic variants leading to SCD encompass this domain [10]. The first loop is most frequently affected by SCD pathogenic variants which appear to disturb its hydrophilic property [14].

The purpose of the study was to report two known and a novel *UBIAD1* gene variant causative of SCD and present a clinical and molecular characterization of the disease in the context of systemic findings in four previously unreported Polish SCD families.

## Patients and methods

### Study subjects

**Fig. 1:**
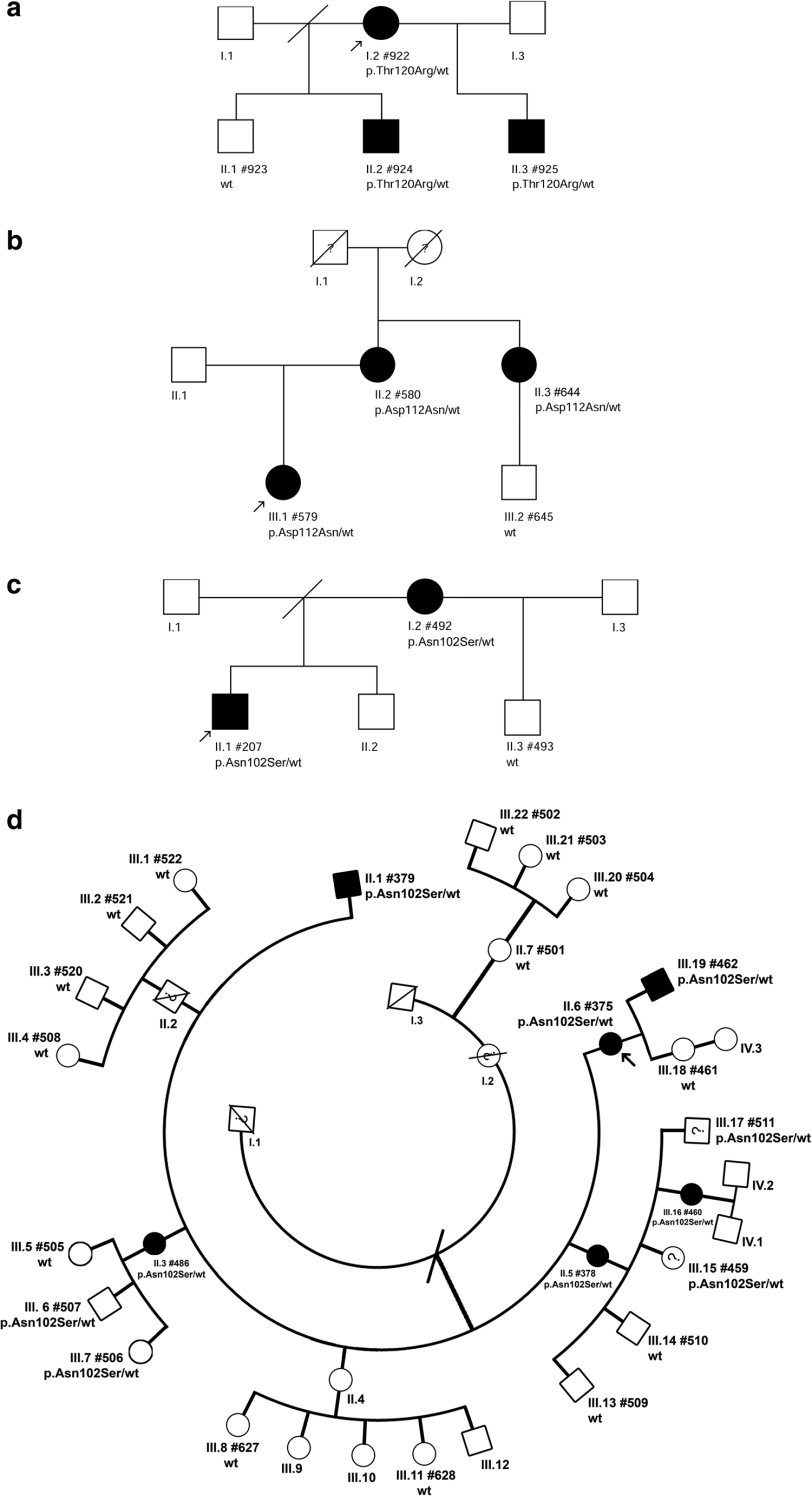
Pedigrees of the analyzed SCD families For every examined patient a corresponding identification number (#PatID format) together with a detected *UBIAD1* allelic variant (wt– wild type, p.Thr120Arg – Pedigree no. 690 (**a**), p.Asp112Asn – Pedigree no. 411 (**b**), p.Asn102Ser – Pedigree nos. 149 and 272 (**c** and **d**)) are shown. Black symbols indicate affected, white symbols unaffected individuals, symbols with a diagonal line indicate deceased individuals, symbols with question mark indicate individuals not examined ophthalmologically, probands are marked with arrows

Blood samples were collected from 37 individuals (14 SCD affected, 21 unaffected, and 2 not examined ophthalmologically) from four Polish families (Ped. nos. 690, 411, 149, 272, Fig. 1a–d), one of them with a three-generation history of SCD (Ped. no. 272, Fig. 1d).

### Ophthalmological evaluation

The subjects underwent complete ophthalmological examinations including uncorrected and best corrected visual acuity (UCVA/BCVA), intraocular pressure measurement, and slit-lamp biomicroscopy. In patients with identified corneal changes, Anterior Segment-OCT (CASIA SS-1000, Tomey, Nagoya, Japan) and in vivo confocal microscopy (CS3/CS4, Nidek Tech., Padova, Italy) were also performed.

### Genetic testing and bioinformatic analysis

Genomic DNA was isolated from blood samples (*n* = 37) with a standard salting-out procedure. DNA pathogenic variant testing was performed by PCR amplifying and Sanger sequencing of the *UBIAD1* gene coding regions (exons 1 and 2). The PCR primer sequences designed using the reference sequence NG_009443.1 and amplification conditions are available upon request. DNA samples were purified with exonuclease I and FastAP thermosensitive alkaline phosphatase (Thermo Fisher Scientific, Waltham, Massachusetts, USA) according to the manufacturer’s protocol, sequenced directly using ABI Prism 377 DNA Sequencer (Thermo Fisher Scientific) and BigDye Terminator v1.1 Cycle Sequencing Kit (Thermo Fisher Scientific) and analyzed with the Variant Reporter DNA analysis software v1.1 (Thermo Fisher Scientific).

Pathogenicity of the novel non-synonymous single nucleotide *UBIAD1* variant was predicted using PredictSNP2 [15], FATHMM [16], and MutPred2 [17], leading and reliable computational approaches [18] and analyzed for population frequency based on the data from Exome Aggregation Consortium (ExAC, http://exac.broadinstitute.org), 1000 Genomes Project (http://www.1000genomes.org), and NHLBI GO Exome Sequencing Project (ESP, http://evs.gs.washington.edu/EVS; all accessed 06/2018).

## Results

### Identified *UBIAD1* gene pathogenic variants in patients with SCD

*UBIAD1* gene pathogenic variants were found in a total of 18 subjects (11 females and 7 males aged 13–68 years; mean age 37.8 y/o). Two of them (III.7 PatID#506 and III.6 PatID#507) did not present signs of SCD, most probably because of their relatively young age (16 and 21 y/o, respectively) or incomplete penetrance of the identified *UBIAD1* variant. In the other two individuals (III.15 PatID#459 and III.17 PatID#511) with severe intellectual disability, a detailed ophthalmological examination could not be conducted (Table 1). The remaining 19 unaffected subjects did not carry any *UBIAD1* disease-causing alteration (Fig. 1a–d).

**Table 1:** Clinical and genetic characterization of SCD patients from this study

Ped. no.	PedID/PatID	UBIAD1 pathogenic variant	Sex	Age at examination	Crystals C/P	Haze C/D	Arcus lipoides	BCVA OD/OS	Other medical conditions
690	I.2/922	p.Thr120Arg	F	44	+ P	−/−	–	0.7/0.5	Hypertension, varicose veins
				51	++++ C/P	+	+	0.2/0.2	
	II.2/924	p.Thr120Arg	M	22	+ P	−/−	–	1.0/0.9	–
	II.3/925	p.Thr120Arg	M	13	+ P	−/−	–	0.7/0.4	intellectual disability, inguinal hernia
411	II.2/580	p.Asp112Asn	F	65	++++ C/P	+	+	0.8/0.7	n/a
	II.3/644	p.Asp112Asn	F	68	++++ C/P	+/+	+	0.4/0.2	↑ cholesterol, hypertension, cholelithiasis
	III.1/579	p.Asp112Asn	F	36	++ C/P	–	–	0.9/0.9	–
149	I.2/492	p.Asn102Ser	F	51	n/a	n/a	n/a	n/a	n/a
	II.1/207	p.Asn102Ser	M	33	++ C	+/−	–	0.4/0.5	–
272	II.1/379	p.Asn102Ser	M	47	+++ C/P	+/−	+	0.4/0.9	↑ cholesterol, hypertension
	II.3/486	p.Asn102Ser	F	49	+ C/P	−/−	–	0.8/0.5	hypoacusis
	II.5/378	p.Asn102Ser	F	54	++++ C/P	+/−	+	0.4/0.5	↑ cholesterol, hypertension
	II.6/375	p.Asn102Ser	F	54	++++ C/P	+/−	+	0.4/0.3	↑ cholesterol
	III.6/507	p.Asn102Ser	M	21	–	–	–	n/a	intellectual disability
	III.7/506	p.Asn102Ser	F	16	–	–	–	n/a	–
	III.15/459	p.Asn102Ser	F	29	n/a	n/a	–	n/a	↑ cholesterol, cholelithiasis, intellectual disability
	III.16/460	p.Asn102Ser	F	31	++ C/P	−/−	–	0.8/0.8	↑ cholesterol
	III.17/511	p.Asn102Ser	M	26	n/a	n/a	–	n/a	intellectual disability
	III.19/462	p.Asn102Ser	M	26	+ P	−/−	–	0.4/0.9	–

*Crystals C/P* central/paracentral, *haze C/D* central/diffused, *sex F/M* female/male, *n/a* not available

**Fig. 2:**
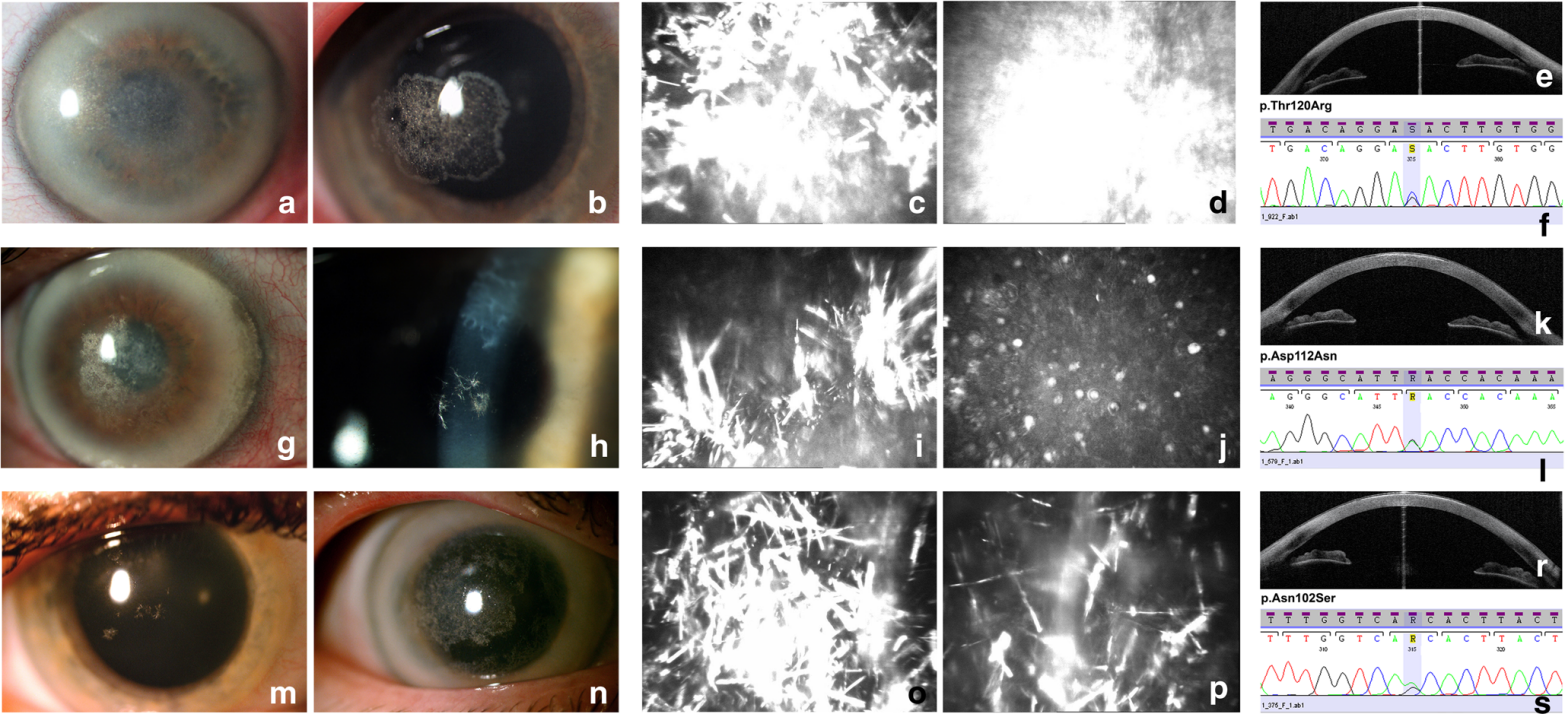
Corneal photographs and electropherograms of the corresponding *UBIAD1* pathogenic variants. First two columns contain slit-lamp photographs showing crystalline formations (**a**, **b**, **g**, **h**, **m**, **n**) in the central and paracentral cornea, arcus lipoides (**a**, **g**, **n**), and haze (**a**, **g**, **n**). Columns three and four contain IVCM images presenting spindle-shaped corneal deposits (**c**, **i**, **o**, **p**), homogeneous conglomerate of deposits (**d**), and microcysts at the epithelial level (**j**). The last column includes AS-OCT images with sagittal sections demonstrating hyperreflective opacities in the anterior part of the corneal stroma (**e**, **k**, **r**). Electropherograms from Sanger sequencing of *UBIAD1* exon 1 showing the identified c.359C > G (p.Thr120Arg) (**f**), c.334G > A (p.Asp112Asn) (**l**), and c.305A > G (p.Asn102Ser) (**s**) pathogenic variants

All pathogenic variants identified in the patients with SCD are located within the first exon of the *UBIAD1* gene. Genetic testing of the first family (Ped. no. 690) shown in Fig. 1a revealed a novel heterozygous missense variant NM_013319.2:c.359C>G predicted to result in amino acid substitution NP_037451.1:p.Thr120Arg (Fig. 2f). The alteration completely segregated with the disease in the family and was predicted to be damaging by PredictSNP2 (score 1.0000, threshold range <0; 1> for pathogenic variants), FATHMM (score 0.8970, threshold range <0.7; 1> for pathogenic variants) and MutPred2 (score 0.8810, threshold range <0.5; 1> for pathogenic variants). The NM_013319.2:c.359C>G transversion has not been reported in population databases.

In patients from the second family (Ped. no. 411), a heterozygous pathogenic variant NM_013319.2:c.334G>A causing a missense change NP_037451.1:p.Asp112Asn was identified (Figs. 1b and 2l). Testing of patients from the third and fourth family (Ped. nos. 149 and 272, Fig. 1c, d) showed the presence of a heterozygous NM_013319.2:c.305A>G pathogenic variant causing the NP_037451.1:p.Asn102Ser amino acid substitution (Fig. 2s) [4, 14].

### Corneal changes in SCD patients from the investigated families

Corneal thickness in all patients was within a normal range, and no signs of corneal edema were observed. All of the symptomatic individuals had corneal crystals.

In family with the novel p.Thr120Arg pathogenic variant (Ped. no. 690), the signs of asymptomatic SCD were identified as early as in the 6 year of age (II.3 PatID#925, Fig. 1a) with the first symptoms occurring at the end of the fourth decade of life (37 y/o; I.2 PatID#922, Fig. 1a). Dystrophic changes were located in the paracentral part of corneal stroma and took form of single crystals. They were more numerous in the proband (44 y/o) than in her two sons (22 and 13 y/o) and the phenotype was assessed as relatively mild. At this time of ophthalmological examination, there was no haze or arcus lipoides in the corneas of the affected individuals (Fig. 2b). AS-OCT images in the affected family members showed dystrophic changes in the form of highly reflective lines of deposits in the anterior and mid-stroma in the central and peripheral part of the cornea (Fig. 2e). IVCM examination in these subjects showed hyperreflective spindle-shaped deposits within the anterior and partly mid-stroma. They were thicker than those observed in patients with p.Asp112Asn and p.Asn102Ser pathogenic variants. Some images revealed diffuse homogenous structures without the spindle-shaped deposits (Fig. 2c, d). During the last 3-year observation period (48–51 y/o) corneal changes in the proband have progressed dramatically from single paracentral crystals to an advanced stage of SCD with arcus lipoides, stromal haze, central corneal opacity, and crystal conglomerates (Fig. 2a). Her BCVA progressed from 0.4 to 0.2. At the age of 51, she has been scheduled for a corneal surgery.

At the time of ophthalmological examination, the proband from family with the *UBIAD1* p.Asp112Asn variant (Ped. no. 411) was 28 y/o and did not present any symptoms of SCD. In her mother, the first symptoms occurred around the age of 60 years. Slit-lamp examination in the affected individuals from this family revealed age-dependent signs of SCD (Fig. 2g, h) [5]. AS-OCT imaging showed hyperreflective deposits penetrating to the deep anterior and middle parts of the stromal layer in the central cornea. At the epithelial side, the upper border of the deposits was slightly irregular (Fig. 2k). IVCM examination revealed the presence of thin, hyperreflective spindle-shaped deposits (Fig. 2i). Some of them conglomerated into characteristic homogeneous substance comparable to dystrophic changes observed in IVCM images of Reis-Bücklers and Thiel-Behnke corneal dystrophy [1]. Additionally, in the basal epithelial layer, single microcysts were found and they were similar to those observed in Meesmann’s corneal dystrophy (Fig. 2j) [19]. At the age of 68, the proband’s mother underwent PKP due to a low VA as a consequence of SCD progression. After 4 years post-transplantation, none of the signs of SCD recurrence in the transplanted cornea has been observed.

The p.Asn102Ser *UBIAD1* pathogenic variant was found in two families (Ped. nos. 149 and 272) with two and six affected individuals, respectively. There was a great variability in the age of symptoms onset in both families, ranging from 9 y/o (II.6 PatID#375) to 51 y/o (II.3 PatID#486). In slit-lamp examination, similar phenotypic features with haze in the central and paracentral parts of the cornea, crystalline formations, and thick yellow-white arcus lipoides in the advanced SCD stages were visible (Fig. 2m, n). AS-OCT revealed highly reflective deposits localized in the anterior stroma of the central and mid-peripheral part of the cornea (Fig. 2r). IVCM showed well-demarcated hyperreflective spindle-shaped deposits which created star-like formations in the stromal layer (Fig. 2o, p). The number of deposits was higher in the advanced stages of SCD and keratocytes could not be visualized.

## Discussion

In this study we have identified p.Thr120Arg, a novel heterozygous point alteration in the *UBIAD1* gene causative of SCD. The second pathogenic variant p.Asp112Asn reported here was previously published by Nickerson et al. [14] in the context of in vitro functional studies. To the best of our knowledge, no detailed clinical characterization of SCD patients with this pathogenic variant has been provided so far. The current study delivers independent evidence for the pathogenic potential of UBIAD1 p.Asp112Asn and reports the genetic variant for the first time in Polish SCD patients. The third *UBIAD1* pathogenic variant detected in the study, p.Asn102Ser, is identified in the majority of unrelated SCD families in different ethnic groups and it is believed that this variant represents a hot spot change for SCD [4].

We have identified the pathogenic variant p.Asn102Ser in two unrelated Polish SCD families in as many as 12 out of 18 genetically confirmed SCD patients. One of the families (Ped. no. 272, Fig. 1d) represents one of the most numerous SCD kindreds, so far reported, for which we have conducted thorough ophthalmological and genetic examinations. Our findings contribute to a previous study describing two other Polish families with SCD as a result of *UBIAD1* p.Asn102Ser [11] and confirm that the pathogenic variant is also the most common genetic alteration found in SCD patients from Central Europe.

The last decade brought the discovery of several fundamental functions of the *UBIAD1*-encoded protein. These encompass (i) synthesis of human endogenous form of vitamin K_2_ (MK-4) from derivates of a plant form vitamin K_1_ [10, 20–25], (ii) prevention of oxidative damage in tissues by synthesis of non-mitochondrial coenzyme Q10 [26, 27], and (iii) direct and indirect interaction with proteins that regulate cholesterol synthesis and transport (HMGCR, SOAT1, apoE) [10, 28, 29]. UBIAD1 protein plays a crucial role in maintaining lipid-cholesterol homeostasis in different cell types [10, 12, 21, 30, 31] but the molecular mechanism by which UBIAD1 pathogenic variants affect the cornea leading to lipid deposition in SCD patients has yet to be determined.

Defective function of UBIAD1 protein results in reduction of the local synthesis of endogenous form of vitamin K_2_ in cells and impairment of cholesterol and lipid metabolism leading to a continuous steroidogenesis stimulation and tissue-specific cholesterol and lipid deposition [10, 29]. Codon p.Thr120 of the UBIAD1 protein is placed directly between two other codons which were previously identified to be altered in SCD — p.Arg119Gly and p.Leu121Phe/Val [10]. All of these amino acids are placed within the first aspartate–rich motif (FARM) which localizes to the first polypeptide loop of the UBIAD1 protein. It is a highly conserved region that may play a crucial role in synthesis of sterols and isoprenoid lipids, as well as cellular cholesterol binding, storage, and transport [10, 12]. Accordingly, p.Thr120Arg along with other SCD causing pathogenic variants is predicted to strongly affect UBIAD1 protein folding and stability, protein enzymatic function, and protein–protein interactions. These alterations may have deleterious impact on cholesterol metabolism in the cornea, contributing to lipid deposition and cholesterol esterification, which may lead to corneal haze and crystalline formation, characteristic features of the SCD phenotype.

Some of our SCD patients reported cardiovascular system disorders (6/18; 33%) and/or cholelithiasis (Table 1). The most frequent systemic finding in SCD is an elevated cholesterol level in blood plasma. Generally, hypercholesterolemia is shown to be present in 66% of patients with SCD [5]. In the 2013–2014 survey on Polish population, the prevalence of hypercholesterolemia averaged 67.3%, (70.3% for men, 64.3% for women) [32]. Occurrence of cholesterol deposits in the cornea is described to show no relation with severity of systemic dyslipidemia [6]. Moreover, SCD corneas present a greater tendency to accumulate high-density lipoproteins (HDL) than low-density lipoproteins (LDL) [33]. Progression of the corneal opacification is also not related to the level of lipids in the blood plasma [5]. It is shown that statin treatment and control of systemic cholesterol do not inhibit the progression of SCD [34].

In line with other reports, we have observed a gradual loss of VA in SCD patients which was progressing along with the severity of dystrophic changes and corresponded with the age of affected individuals [5, 6, 14, 35]. Unlike the moderate severity stage of SCD, which is usually recognized on the basis of slit-lamp biomicroscopy, initial and advanced stages of the disease tend to cause a more significant diagnostic problem. In the early SCD stage, the signs can be easily overlooked and in the advanced stage, fused corneal opacities and stromal deposits may resemble other corneal dystrophies or corneal degeneration. The majority of our patients presented a moderate severity stage of SCD and slit-lamp examination demonstrated a characteristic clinical picture of the dystrophy. Interestingly, at the end of the fifth decade of life in the proband with *UBIAD1* p.Thr120Arg initially mild dystrophic changes progressed rapidly from an early to advanced SCD stage only within a 3-year observation period. It is a quite unusual finding as SCD generally progresses gradually [5, 6, 14, 35].

In general, the appearance of deposits, their reflectance, location, and the images of corneal epithelium and endothelium in IVCM imaging in our patients is in line with the descriptions by other authors [33, 36–38]. However, in patients with p.Asp112Asn, we also observed small cysts with hyperreflective content in the corneal epithelium (Fig. 2j); such changes are only rarely observed in SCD patients [11, 39]. To the best of our knowledge, there are only two other reports on SCD visualized by AS-OCT [11, 40]. Both studies described stromal hyperreflective opacities limited to the anterior parts of the cornea corresponding with the localization of crystalline formation visible in IVCM images, which is consistent with our observations. In AS-OCT, the appearance of corneal changes was similar in all subjects, but the quantity of deposits was noticeably different and appropriate to SCD stage.

Corneal imaging with IVCM and AS-OCT is proven to be helpful in differential diagnosis of inapparent SCD cases. However, IVCM may not be a conclusive approach as corneal crystalline formations in SCD are similar to those observed, e.g., in cystinosis or infectious crystalline keratopathy. Along with the increasing availability of genetic testing, identification of an *UBIAD1* pathogenic variant has become a necessary complement to ophthalmological examinations as it provides a definitive confirmation of clinical SCD diagnosis.

## References

[1] Weiss JS, Moller HU, Aldave AJ, Seitz B, Bredrup C, Kivela T, Munier FL, Rapuano CJ, Nischal KK, Kim EK, Sutphin J, Busin M, Labbe A, Kenyon KR, Kinoshita S, Lisch W (2015). IC3D classification of corneal dystrophies--edition 2. Cornea.

[2] Kim EK, Lee H, Choi SI (2015). Molecular pathogenesis of corneal dystrophies: Schnyder dystrophy and granular corneal dystrophy type 2. Prog Mol Biol Transl Sci.

[3] Orr A, Dube MP, Marcadier J, Jiang H, Federico A, George S, Seamone C, Andrews D, Dubord P, Holland S, Provost S, Mongrain V, Evans S, Higgins B, Bowman S, Guernsey D, Samuels M (2007). Mutations in the UBIAD1 gene, encoding a potential prenyltransferase, are causal for Schnyder crystalline corneal dystrophy. PLoS One.

[4] Weiss JS, Kruth HS, Kuivaniemi H, Tromp G, White PS, Winters RS, Lisch W, Henn W, Denninger E, Krause M, Wasson P, Ebenezer N, Mahurkar S, Nickerson ML (2007). Mutations in the UBIAD1 gene on chromosome short arm 1, region 36, cause Schnyder crystalline corneal dystrophy. Invest Ophthalmol Vis Sci.

[5] Weiss JS (2009). Schnyder corneal dystrophy. Curr Opin Ophthalmol.

[6] Weiss JS, Khemichian AJ (2011). Differential diagnosis of Schnyder corneal dystrophy. Dev Ophthalmol.

[7] Weiss JS (2016). The Oskar Fehr lecture. Klin Monatsbl Augenheilkd.

[8] Chae H, Kim M, Kim Y, Kim J, Kwon A, Choi H, Park J, Jang W, Lee YS, Park SH, Kim MS (2016). Mutational spectrum of Korean patients with corneal dystrophy. Clin Genet.

[9] Lin BR, Frausto RF, Vo RC, Chiu SY, Chen JL, Aldave AJ (2016). Identification of the first de novo UBIAD1 gene mutation associated with Schnyder corneal dystrophy. J Ophthalmol.

[10] Nickerson ML, Bosley AD, Weiss JS, Kostiha BN, Hirota Y, Brandt W, Esposito D, Kinoshita S, Wessjohann L, Morham SG, Andresson T, Kruth HS, Okano T, Dean M (2013). The UBIAD1 prenyltransferase links menaquinone-4 [corrected] synthesis to cholesterol metabolic enzymes. Hum Mutat.

[11] Nowinska AK, Wylegala E, Teper S, Lyssek-Boron A, Aragona P, Roszkowska AM, Micali A, Pisani A, Puzzolo D (2014). Phenotype-genotype correlation in patients with Schnyder corneal dystrophy. Cornea.

[12] Fredericks WJ, McGarvey T, Wang H, Lal P, Puthiyaveettil R, Tomaszewski J, Sepulveda J, Labelle E, Weiss JS, Nickerson ML, Kruth HS, Brandt W, Wessjohann LA, Malkowicz SB (2011). The bladder tumor suppressor protein TERE1 (UBIAD1) modulates cell cholesterol: implications for tumor progression. DNA Cell Biol.

[13] Hirota Y, Nakagawa K, Sawada N, Okuda N, Suhara Y, Uchino Y, Kimoto T, Funahashi N, Kamao M, Tsugawa N, Okano T (2015). Functional characterization of the vitamin K2 biosynthetic enzyme UBIAD1. PLoS One.

[14] Nickerson ML, Kostiha BN, Brandt W, Fredericks W, Xu KP, Yu FS, Gold B, Chodosh J, Goldberg M, Lu DW, Yamada M, Tervo TM, Grutzmacher R, Croasdale C, Hoeltzenbein M, Sutphin J, Malkowicz SB, Wessjohann L, Kruth HS, Dean M, Weiss JS (2010). UBIAD1 mutation alters a mitochondrial prenyltransferase to cause Schnyder corneal dystrophy. PLoS One.

[15] Bendl J, Musil M, Stourac J, Zendulka J, Damborsky J, Brezovsky J (2016). PredictSNP2: a unified platform for accurately evaluating SNP effects by exploiting the different characteristics of variants in distinct genomic regions. PLoS Comput Biol.

[16] Shihab HA, Gough J, Cooper DN, Stenson PD, Barker GL, Edwards KJ, Day IN, Gaunt TR (2013). Predicting the functional, molecular, and phenotypic consequences of amino acid substitutions using hidden Markov models. Hum Mutat.

[17] Pejaver V, Urresti J, Lugo-Martinez J, Pagel KA, Lin GN, Nam H-J, Mort M, Cooper DN, Sebat J, Iakoucheva LM, Mooney SD, Radivojac P (2017) MutPred2: inferring the molecular and phenotypic impact of amino acid variants. bioRxiv. 10.1101/134981 10.1038/s41467-020-19669-x PMC7680112 33219223

[18] Walters-Sen LC, Hashimoto S, Thrush DL, Reshmi S, Gastier-Foster JM, Astbury C, Pyatt RE (2015). Variability in pathogenicity prediction programs: impact on clinical diagnostics. Mol Genet Genomic Med.

[19] Szaflik JP, Oldak M, Maksym RB, Kaminska A, Pollak A, Udziela M, Ploski R, Szaflik J (2008). Genetics of Meesmann corneal dystrophy: a novel mutation in the keratin 3 gene in an asymptomatic family suggests genotype-phenotype correlation. Mol Vis.

[20] Hegarty JM, Yang H, Chi NC (2013). UBIAD1-mediated vitamin K2 synthesis is required for vascular endothelial cell survival and development. Development.

[21] Liu S, Guo W, Han X, Dai W, Diao Z, Liu W (2016). Role of UBIAD1 in intracellular cholesterol metabolism and vascular cell calcification. PLoS One.

[22] Nakagawa K, Hirota Y, Sawada N, Yuge N, Watanabe M, Uchino Y, Okuda N, Shimomura Y, Suhara Y, Okano T (2010). Identification of UBIAD1 as a novel human menaquinone-4 biosynthetic enzyme. Nature.

[23] Nakagawa K, Sawada N, Hirota Y, Uchino Y, Suhara Y, Hasegawa T, Amizuka N, Okamoto T, Tsugawa N, Kamao M, Funahashi N, Okano T (2014). Vitamin K2 biosynthetic enzyme, UBIAD1 is essential for embryonic development of mice. PLoS One.

[24] Shearer MJ, Fu X, Booth SL (2012). Vitamin K nutrition, metabolism, and requirements: current concepts and future research. Adv Nutr.

[25] Shearer MJ, Newman P (2014). Recent trends in the metabolism and cell biology of vitamin K with special reference to vitamin K cycling and MK-4 biosynthesis. J Lipid Res.

[26] Li W (2016). Bringing bioactive compounds into membranes: the UbiA superfamily of intramembrane aromatic Prenyltransferases. Trends Biochem Sci.

[27] Mugoni V, Postel R, Catanzaro V, De Luca E, Turco E, Digilio G, Silengo L, Murphy MP, Medana C, Stainier DY, Bakkers J, Santoro MM (2013). Ubiad1 is an antioxidant enzyme that regulates eNOS activity by CoQ10 synthesis. Cell.

[28] Schumacher MM, Jun DJ, Jo Y, Seemann J, DeBose-Boyd RA (2016). Geranylgeranyl-regulated transport of the prenyltransferase UBIAD1 between membranes of the ER and Golgi. J Lipid Res.

[29] Schumacher MM, Elsabrouty R, Seemann J, Jo Y, DeBose-Boyd RA (2015) The prenyltransferase UBIAD1 is the target of geranylgeraniol in degradation of HMG CoA reductase. eLife. 10.7554/eLife.05560 10.7554/eLife.05560 PMC4374513 25742604

[30] Fredericks WJ, Sepulveda J, Lai P, Tomaszewski JE, Lin MF, McGarvey T, Rauscher FJ, Malkowicz SB (2013). The tumor suppressor TERE1 (UBIAD1) prenyltransferase regulates the elevated cholesterol phenotype in castration resistant prostate cancer by controlling a program of ligand dependent SXR target genes. Oncotarget.

[31] Fredericks WJ, Yin H, Lal P, Puthiyaveettil R, Malkowicz SB, Fredericks NJ, Tomaszewski J, Rauscher FJ, Malkowicz SB (2013). Ectopic expression of the TERE1 (UBIAD1) protein inhibits growth of renal clear cell carcinoma cells: altered metabolic phenotype associated with reactive oxygen species, nitric oxide and SXR target genes involved in cholesterol and lipid metabolism. Int J Oncol.

[32] Pajak A, Szafraniec K, Polak M, Polakowska M, Kozela M, Piotrowski W, Kwasniewska M, Podolecka E, Kozakiewicz K, Tykarski A, Zdrojewski T, Drygas W, Investigators W (2016). Changes in the prevalence, treatment, and control of hypercholesterolemia and other dyslipidemias over 10 years in Poland: the WOBASZ study. Pol Arch Med Wewn.

[33] Gaynor PM, Zhang WY, Weiss JS, Skarlatos SI, Rodrigues MM, Kruth HS (1996). Accumulation of HDL apolipoproteins accompanies abnormal cholesterol accumulation in Schnyder’s corneal dystrophy. Arterioscler Thromb Vasc Biol.

[34] Lisch W, Weidle EG, Lisch C, Rice T, Beck E, Utermann G (1986). Schnyder’s dystrophy. Progression and metabolism. Ophthalmic Paediatr Genet.

[35] Weiss JS (1992). Schnyder’s dystrophy of the cornea. A Swede-Finn connection. Cornea.

[36] Mastropasqua LNM (2002). Basic principles of confocal microscopy of the cornea.

[37] Vesaluoma MH, Linna TU, Sankila EM, Weiss JS, Tervo TM (1999). In vivo confocal microscopy of a family with Schnyder crystalline corneal dystrophy. Ophthalmology.

[38] Kobayashi A, Fujiki K, Murakami A, Sugiyama K (2009). In vivo laser confocal microscopy findings and mutational analysis for Schnyder’s crystalline corneal dystrophy. Ophthalmology.

[39] Arnold-Worner N, Goldblum D, Miserez AR, Flammer J, Meyer P (2012). Clinical and pathological features of a non-crystalline form of Schnyder corneal dystrophy. Graefes Arch Clin Exp Ophthalmol.

[40] Jing Y, Liu C, Xu J, Wang L (2009). A novel UBIAD1 mutation identified in a Chinese family with Schnyder crystalline corneal dystrophy. Mol Vis.

